# Recent Progress in Self-Powered Wireless Sensors and Systems Based on TENG

**DOI:** 10.3390/s23031329

**Published:** 2023-01-24

**Authors:** Yonghai Li, Jinran Yu, Yichen Wei, Yifei Wang, Zhenyu Feng, Liuqi Cheng, Ziwei Huo, Yanqiang Lei, Qijun Sun

**Affiliations:** 1 Center on Nanoenergy Research, School of Chemistry and Chemical Engineering, Guangxi University, Nanning 530004, China; 2 Beijing Institute of Nanoenergy and Nanosystems, Chinese Academy of Sciences, Beijing 101400, China; 3 School of Nanoscience and Technology, University of Chinese Academy of Sciences, Beijing 100049, China

**Keywords:** self-powered sensors, wireless sensors, energy harvesting, triboelectric nanogenerator

## Abstract

With the development of 5G, artificial intelligence, and the Internet of Things, diversified sensors (such as the signal acquisition module) have become more and more important in people’s daily life. According to the extensive use of various distributed wireless sensors, powering them has become a big problem. Among all the powering methods, the self-powered sensor system based on triboelectric nanogenerators (TENGs) has shown its superiority. This review focuses on four major application areas of wireless sensors based on TENG, including environmental monitoring, human monitoring, industrial production, and daily life. The perspectives and outlook of the future development of self-powered wireless sensors are discussed.

## 1. Introduction

In recent years, human daily life has been greatly improved with the rapid development of the world’s economy and technology [1]. In particular, the development of the Internet of Things (IoT), artificial intelligence (AI), and electronic equipment has enabled us to obtain and process massive amounts of information in real time, which greatly enhances our ability to perceive the world [2,3,4,5]. Thus, sensor technology and Wireless Sensor Networks (WSNs) containing many sensors are becoming more and more important and have attracted much attention from researchers and industries [6]. Traditional sensors transmit the collected data to the post-processor by wires, which was normal and efficient in the industrial age. However, as the world shifts into the age of AI and the IoT, the range of human activities is expanding everywhere in the world (and even to Mars), which greatly boosts the use of wireless sensors. A wireless sensor, which is portable and remotely controllable, can function in volcanic craters, long-distance transmission lines, oil or gas pipelines, long-distance optical cables, forests, oceans, or other places where humans cannot work for extended periods of time [7]. The use of wireless sensors makes the IoT a reality and provides fundamental data for AI, which constructs a digital world that serves humans.

Powering these wireless sensors has become an urgent problem when they are used extensively. Until now, the most frequently used power source is a battery, which stores a given amount of electric energy and can power the sensor for a certain duration. When the electrical energy stored in the battery is used, the battery must be recharged or replaced, which is an insupportable burden for the increasing number of sensors. The other challenge of using batteries is pollution originating from the electrolytes contained in the thousands of tons of used batteries [8,9,10,11]. To avoid these issues, self-powered sensors provide an effective solution, which can harvest energy from surroundings (such as light, heat, and mechanical vibration) to power themselves. 

A nanogenerator is generally considered the ideal real-time power supply for wireless sensors. Since the triboelectric nanogenerator (TENG) was invented by Wang’s group in 2012, many researchers have proved the TENG is a novel, feasible, and effective device to harvest energy from the ambient environment [8,12,13,14]. As a new self-powered sensing technology, TENGs are aptly used in the self-powered wireless sensing field [15,16,17,18]. 

**Figure 1:**
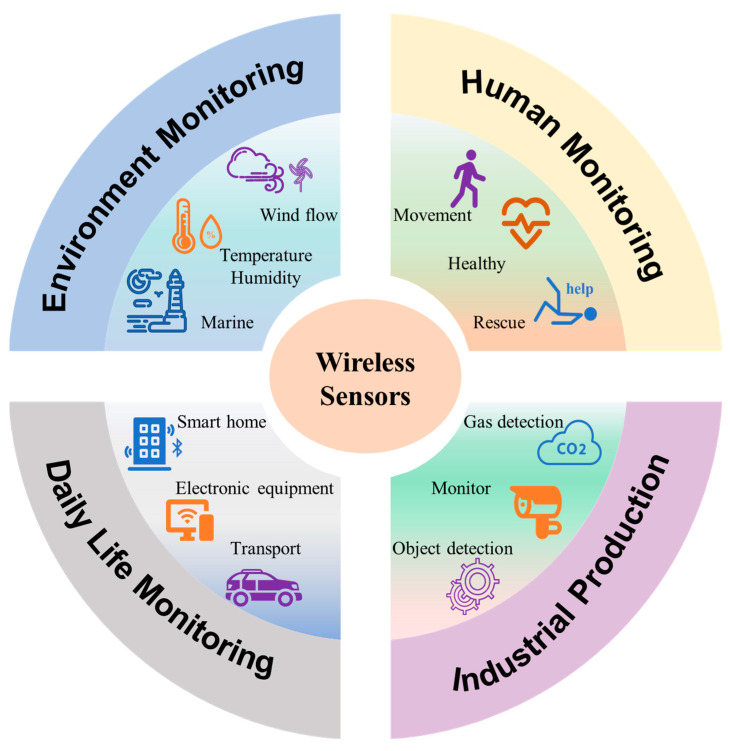
Schematic illustration of wireless sensors for environmental monitoring, human monitoring, industrial production, and daily life.

The aim of this paper is to review the applications of wireless sensors based on the TENG in environmental monitoring, human monitoring, industrial application, and daily life, which are the four most important parts of human civilization today and are graphically summarized in Figure 1. Firstly, a brief introduction is given in Section 1 to present the advantages of wireless sensors based on the TENG for its inherent self-powered characteristics. Then, the physical origin and working principles of the TENG are briefly introduced in Section 2. In Section 3, the applications of a wireless sensor based on TENG in different fields, including environmental monitoring, human monitoring, industrial protection, and daily life, are described in detail. At last, a summary and outlook on self-powered wireless sensors based on the TENG are provided to discuss the current challenges, which may pave the way to the better development of self-powered wireless sensors in the future.

## 2. The Principles of TENG

After Wang’s group presented the TENG for the first time, which can convert ambient chaotic low-frequency mechanical vibration into electrical energy [8,19,20,21,22,23,24,25,26,27], the working principles of the TENG were gradually established from both experimental and theoretical perspectives [28,29,30,31,32,33,34,35].

### 2.1. Triboelectrification Effect 

With the development of the world, the understanding of triboelectrification is deepening, and the mechanism of electrostatic induction has been gradually proposed [36]. Triboelectrification (TE) and contact electrification (CE) are natural physical phenomena and we generally consider TE as CE [34]. With the continuous exploration and understanding of friction electrification, scientists find that TE and CE are not the same physical phenomenon, and they have significant differences. CE only occurs through physical contact between two materials, while TE usually involves friction between two materials [33]. 

Triboelectrification (TE) is usually interpreted as the phenomenon that two different materials are charged when contacting and separating [37]. As is known, the Fermi level of a conductor determines the thermodynamic work needed to remove an electron from it to a point in the vacuum immediately outside its surface [38]. The contact potential difference (CPD) is generally defined as the Fermi energy difference between two contact materials. When two different materials come into contact with each other, CPD can drive the transfer of electrons from a high energy state (valence band) on one surface to a lower energy state (conduction band) on the other surface. [39,40]. As Duke et al. discussed, the charges transferred to the insulator surface are confined and concentrated there instead of passing immediately when conductor–insulator contact or insulator–insulator contact is constructed. Their polarities depend entirely on the direction of electron flow during contact (which depends on the polarity of the CPD of the two contacting materials) [41]. This process of accumulating charge on the surface of an insulator is known as contact electrification [36]. 

### 2.2. Theoretical Models of TENGs

The basic principles of TENGs are derived from Maxwell’s displacement currents [39]. This current is generated from the inside of the friction material, while electrons are constantly flowing back and forth in an external circuit between the two electrodes. As the triboelectric voltage drives the electron flow, the inner and outer circuits intersect at the two electrodes and the entire transport process is integrated. Displacement currents are inherent to the physical core of TENG.

Maxwell’s displacement current [42] is expressed as

(1) $$J_{D}=\frac{\partial D}{\partial t}=\varepsilon_{0}\frac{\partial E}{\partial t}+\frac{\partial P}{\partial t}$$

where *E* denotes the electric field, *D* denotes the displacement field, and *P* denotes the polarization field. The first component ($\varepsilon_{0}\frac{\partial E}{\partial t}$) in this equation is the theoretical basis of an alternating-current generator and wireless communication. This part predicted the existence of an electromagnetic wave and laid a physical foundation for radio communication. The second part ($\;\frac{\partial P}{\partial t}$) is closely related to the output signals of the nanogenerator, which is the theoretical basis and source of the TENG and has made great contributions to energy harvesting and self-powered sensors.

The expanded Maxwell equations [43] are expressed as

(2) $$▽\cdot D^{\prime }=\rho_{f}+▽\cdot P_{s}$$

(3) ▽·D=0

(4) $$▽\times E=-\frac{\partial B}{\partial t}$$

(5) $$▽\times H=J_{f}+\frac{\partial D^{\prime }}{\partial t}+\frac{\partial P_{s}}{\partial t}$$

In the above equation, *D* = $D^{\prime }$ + $P_{s}$ = $\varepsilon_{0}$*E* + *P* + $P_{s}$, $\rho_{f}$ denotes the free electric charge density. It is emphasized here that *P_s_* denotes the mechano-driving-created polarization owing to the relative movement of the charged media surfaces as a result of mechanical triggering, *B* denotes the magnetic field, and *J_f_* denotes the free electric current density [44].

The expanded Maxwell equations are referred to as the general theory of TENGs to explain how mechanical energy can be converted into electricity by using displacement current as a driving force to effectively convert mechanical energy into electrical energy [43,44].

### 2.3. Operation Modes of TENGs

**Figure 2:**
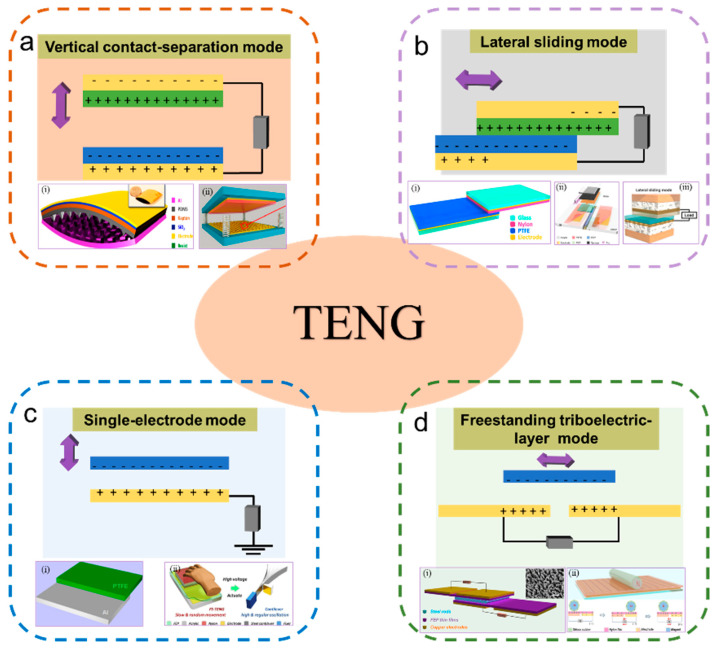
The four fundamental modes of triboelectric nanogenerators and several typical cases of them. (**a**) Vertical contact–separation mode. Reproduced with permission [19]. Copyright 2012, American Chemical Society. Reproduced with permission [20]. Copyright 2013, American Chemical Society. (**b**) Lateral sliding mode. Reproduced with permission [21]. Copyright 2013, American Chemical Society. Reproduced with permission [22]. Copyright 2022, John Wiley & Sons. Reproduced with permission [23]. Copyright 2022, Elsevier Ltd. (**c**) Single-electrode mode. Reproduced with permission [24]. Copyright 2013, American Chemical Society. Reproduced with permission [25]. Copyright 2019, Elsevier Ltd. (**d**) Freestanding triboelectric-layer mode. Reproduced with permission [26]. Copyright 2015, American Chemical Society. Reproduced with permission [27]. Copyright 2019, Elsevier Ltd.

Since the TENG was first reported in 2012, four basic working modes have been proposed according to the direction of the friction electrode and electrode structure, including the vertical contact–separation mode, lateral sliding mode, single-electrode mode, and freestanding triboelectric-layer mode (Figure 2).

The vertical contact–separation mode adopts a configuration in which two different materials are opposite to each other and two electrodes are deposited on the top and bottom surfaces of the stacked structures (Figure 2a). At the initial stage of contact between two friction layers, an equal number of opposite charges will be formed on the contact surface due to contact electrification. It is important to emphasize that the positive and negative charges on the surface of the materials depend on the relative electronegativity of the friction layer. When the two friction layers begin to separate, a small gap will be formed between the two different materials. At the same time, the inductive potential is formed between the two electrodes. When the two electrodes are connected by a load, electrons will flow from one electrode to the other, forming a reverse potential to balance the electrostatic field. When the two friction materials return to the original state, the induced potential disappears, and the electrons flow back. In these four modes, the vertical contact separation mode is the first mentioned and exhibits many benefits, such as a simple structure, good robustness, high instantaneous power density, etc. [37,45].

The contact-sliding mode is also primarily composed of two different dielectric materials, with two electrodes deposited on the top and bottom surfaces of the stacked structure (Figure 2b). It should be noted that CE and TE work together in this mode. The two different media materials will produce lateral relative displacement under the action of horizontal force. Similar to the vertical contact—separation mode, the contact-sliding mode has the same initial structure. Reciprocating the relative sliding of the two different materials will induce the reversible flow of electrons and an alternating current (AC) output that can be achieved through the external circuit.

The electrodes of the two types of TENGs mentioned above are connected by an external circuit. Unlike the two TENG modes presented above, the single-electrode mode structure has only one electrode connected to the ground (Figure 2c). In this single-electrode model, this sole electrode is used not only as an electrode to conduct electrons but also as a friction material. According to the principle of electrostatic induction, triboelectric charges will be generated on the surface of dielectric materials when they are in contact with single electrodes. Depending on the change in frictional potential during contact separation, the generated electrostatic charge will drive the flow of electrons between the ground and the electrode. The single-electrode model offers many advantages, such as the ability of TENGs to operate freely, a simpler structural design, and more efficient sensing performance. This single-electrode mode allows some designs to be more accessible so that they can be adapted to complex transportation situations and variable environments. Another TENG mode was designed based on the single-electrode mode. The independent triboelectric layer mode is shown in Figure 2d, which uses a pair of symmetrical electrodes as the reference electrode in addition to the ground [46].

## 3. The Application of a Self-Powered Wireless Sensor Based on TENG

Self-powered wireless sensors based on the TENG have been used in various conditions due to their flexibility, sustainability, and affordability. Here, we focus on four fields, i.e., environmental monitoring, human monitoring, industrial application, and daily life.

### 3.1. Environmental Monitoring

The detection of the meteorological environment is of great significance to people’s lives because various disasters (e.g., drought, high temperatures, cold waves, floods, mountain torrents, typhoons, tornados, hail, rainstorms, snowstorms, earthquakes, tsunamis, mud rock flow, sandstorms, lightning, thunderstorms, and volcanic eruptions) have posed great threats to human beings [8,9]. For a better life, human beings need to fight against natural disasters and predict them as early as possible [47]. Therefore, real-time environmental monitoring of the field environment using wireless self-powered sensors is important for human beings.

#### 3.1.1. Meteorological Environment Monitoring 

Wind sensors play a very large role in environmental sensors. With the development of the TENG, many researchers have studied the sensory systems based on the TENG for wind speed measurement [48,49,50,51,52]. In this section, we provide several examples of self-powered wireless wind sensors based on the TENG. 

**Figure 3:**
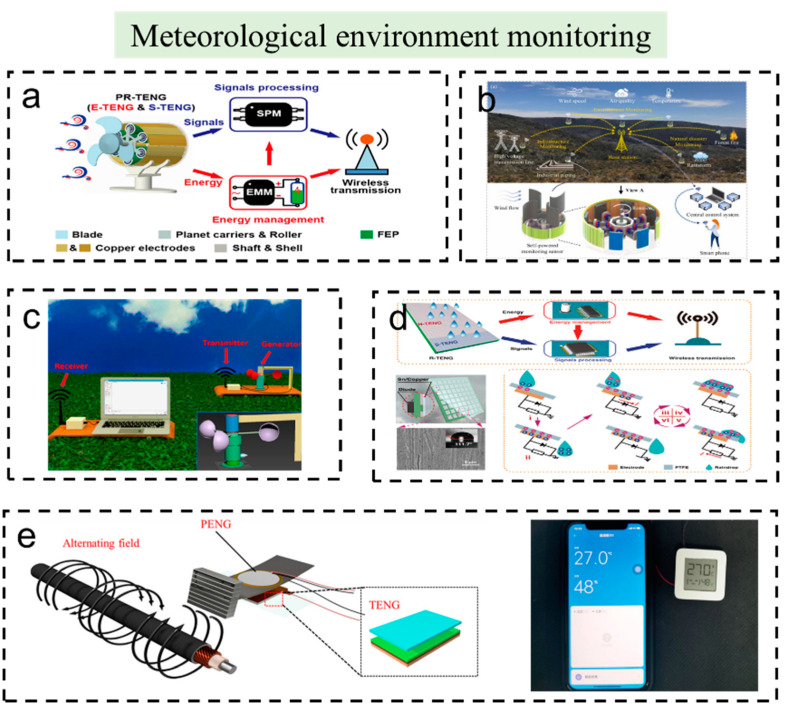
The self-powered wireless sensors and systems based on TENG used in meteorological environment monitoring. (**a**) A breeze-wind-driven autonomous wireless anemometer based on rolling contact electrification. Reproduced with permission [53]. Copyright 2021, American Chemical Society. (**b**) A self-regulation strategy for triboelectric nanogenerator and a self-powered wind-speed sensor. Reproduced with permission [9]. Copyright 2022, Elsevier Ltd. (**c**) A self-powered, wireless, remote meteorologic monitoring system based on an innovative TENG. Reproduced with permission [47]. Copyright 2016, American Chemical Society. (**d**) A raindrop energy-powered autonomous rainfall monitoring and wireless transmission system. Reproduced with permission [56]. Copyright 2022, Springer Nature. (**e**) A hybrid piezo/triboelectric nanogenerator for stray magnetic energy harvesting and a system for temperature and humidity monitoring. Reproduced with permission [57]. Copyright 2021, John Wiley & Sons.

Figure 3a shows a breeze-wind-driven autonomous wireless anemometer (W-WA) based on a planetary rolling triboelectric nanogenerator (PR-TENG), which was reported by Zhang et al. [53]. The TENG not only collects wind energy but also serves as a wind-speed sensor. A PR-TENG can be activated and functionalize at wind speeds below 2 m/s. At wind speeds of 5 m/s, the generated voltage can be maintained above 3.3 volts and the W-WA can be powered continuously and will automatically transmit wind speed data within a 10 m range every 2 min. This work implements a complete self-powered smart wireless sensing system, showing that it is feasible to integrate TENG-based micro/nanoenergy harvesters and an active sensor. The authors mention that self-powered smart wireless sensing systems show great promise for distributed micro/nanoenergy, unattended environmental monitoring, and the Internet of Things. Another breeze sensor system using a self-regulation strategy for a triboelectric nanogenerator (TENG-SS) is shown in Figure 3b [9]. The system is easy to activate and can be adjusted automatically for different wind speeds. The voltage of TENG-SS can increase up to 558 V and the average power is 2.79 mW with d = 0.021 m and m = 0.72 g at the wind speed of 12 m/s. Furthermore, the TENG in this system also has the advantages of minimal wear and good durability. The materials of the TENG with the self-regulation strategy show almost no obvious damage after 21,600 s of continuous testing, showing its superiority in self-powered wireless environment detection. Figure 3c shows a self-powered wireless meteorological monitoring system containing a single-electrode cylindrical TENG and a wind sensor [47]. This system can be placed in remote regions to monitor environmental and ecological information. To avoid the influence of humidity, a humidity-resisting and wind-direction-adapting flag-type TENG for wind energy harvesting and speed sensing was developed [54]. This device was designed as a flag type, and the triboelectric layers are isolated from the air, so the device is hardly affected by humidity. The device has proven that it has great potential applications in wind energy harvesting and self-powered wind speed and direction sensing in humidity conditions. Fang et al. reported a wind energy harvester that can be used as a wind speed sensor itself by analyzing the output frequency characteristics. In addition, the TENG can power a commercial sensor, which can collect temperature and humidity information from the environment. This system enables self-powered wireless environmental monitoring [52].

Similarly, the rain gauge, which is usually used in unattended environments for rainfall monitoring, is a significant device in environmental sensors. Traditional rain gauges have some disadvantages, such as complex mechanical structures, high maintenance costs, and environmental pollution [55]. Xu et al. reported a new rain gauge system, which was powered by raindrops (shown in Figure 3d) [56]. The system can harvest the raindrop energy and serve as a rainfall sensor. The raindrop-TENG array has exhibited good electrical performance. At a rainfall intensity of 71 mm/min, the short-circuit current, open-circuit voltage, and maximum output power of the raindrop-TENG array can reach 15 μA, 1800 V, and 325 μW, respectively. The wireless transmission system can be continuously powered and autonomously transmit rainfall data every 4 min. The authors predict that this work has broad prospects in unattended weather monitoring, field surveys, and the Internet of Things.

Many TENGs are designed to power other sensors, and the collected signals are transmitted to the receiving device through Bluetooth or other modules, realizing the self-powered wireless environment sensor system. Figure 3e shows a hybrid piezoelectric/triboelectric nanogenerator based on a cantilever beam that exploits the stray magnetic field of the ambient environments [57]. The P/TENG delivers high open-circuit voltage (176 V), short-circuit current (375 μA), and maximum output power of ∼4.7 mW under a 4 Oe alternating-current magnetic field induced by a Helmholtz coil. After rectification, the device can provide a stable direct current output with a voltage of 3.6 V, which can sustainably drive commercial wireless temperature/humidity sensors. Roh et al. optimized the structure of a propeller-TENG and obtained a high-power density of 283 mW/m^2^, which can easily power wireless sensor systems composed of commercial temperature and humidity sensors [58]. The energy collected by the propeller-TENG is stored in a capacitor, which powers a commercial temperature and humidity sensor, sending its processed signal to a smartphone via a Bluetooth module. 

#### 3.1.2. Marine Environmental Monitoring

The ocean accounts for approximately 71% of the earth’s area [59]. It is known that the marine ecosystem is the most important ecosystem in the world, which affects the stability and security of the global ecosystem. Therefore, it is extremely important to protect the marine ecological environment when human activities are closely related to the ocean day by day. However, the marine environment is complex and changeable, which makes it difficult to monitor. Wireless sensors constitute one of the most important devices for collecting data and transferring control signals from the marine environment [60]. In recent years, due to the outstanding advantages of TENG, the application of self-powered sensors based on TENGs in marine ecological monitoring has been gradually increasing [61,62,63,64,65]. 

**Figure 4:**
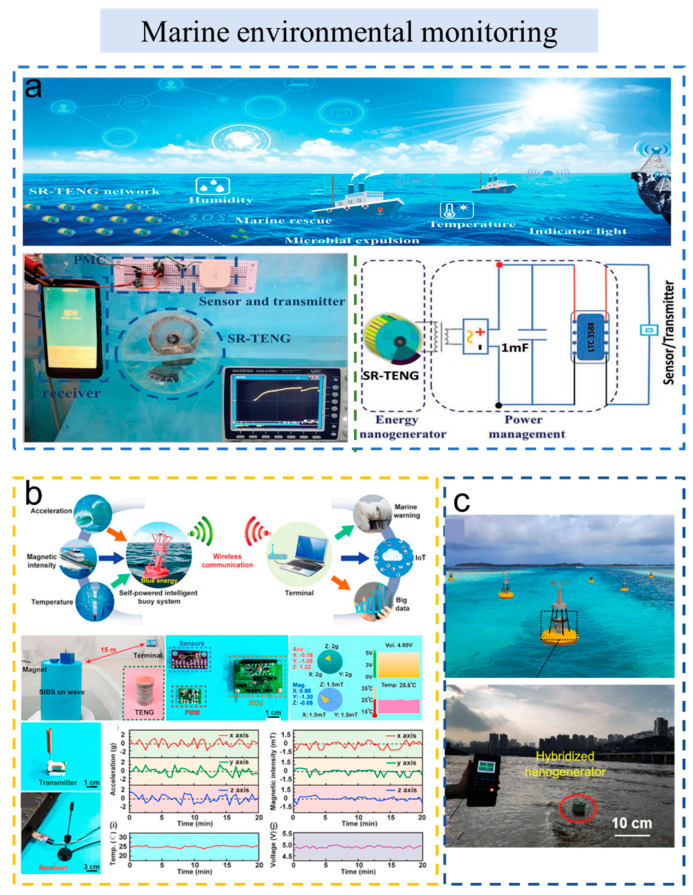
The self-powered wireless sensors and systems based on TENG used in marine environmental monitoring. (**a**) A self-powered and wireless hydrological information monitoring system. Reproduced with permission [66]. Copyright 2022, John Wiley & Sons. (**b**) Self-powered intelligent buoy system by water wave energy for sustainable and autonomous wireless sensing and data transmission. Reproduced with permission [67]. Copyright 2019, Elsevier Ltd. (**c**) A chaotic pendulum triboelectric–electromagnetic hybridized nanogenerator for wave energy scavenging and self-powered wireless sensing system. Reproduced with permission [68]. Copyright 2020, Elsevier Ltd.

As shown in Figure 4a, Cao et al. developed a TENG activated by an oscillating rotary switch mechanism (SR-TENG) [66]. The device can harvest low-frequency mechanical energy from 0.3 to 5 Hz and reach a peak power density of 10.1 W m^−3^ at 1.8 Hz and 15.4 W m^−3^ at 5 Hz, respectively. Finally, they successfully simulated the application of the self-powered wireless system on the night-direction indication, marine lighting and rescue, the expulsion of microorganisms, and ambient temperature/humidity detection. 

Xi et al. reported a self-powered Intelligent Buoy System (SIBS), in which a multi-tiered TENG with high output was used for water wave energy collection, as shown in Figure 4b [67]. Under the wave frequency of 2 Hz, the average output power density of SIBS reaches 13.2 mW/m^2^, allowing this system to realize continuous and independent wireless sensing of acceleration, magnetic strength, and temperature within the range of 15 m, and transmitting 19 bytes every 30 s. This work has provided a common platform for self-powered wireless sensor network nodes and shows great promise for the Internet of Things, big data, artificial intelligence, and blue energy.

A chaotic pendulum triboelectric-electromagnetic hybridized nanogenerator provides a new direction to scavenge low-frequency vibrations from the ocean (as shown in Figure 4c) [68]. In this work, the TENG and electromagnetic nanogenerator (EMG) harvest wave energy to power the wireless sensing nodes with a maximum output power of 15.21 μW and 1.23 mW, respectively. This self-powered wireless sensing node realized distant data transmission exceeding 300 m. Hao et al. also demonstrated a pendulum-type hybrid nanogenerator for scavenging wave energy and powering the hydrophone [69]. The presented swing structure can effectively collect the random water wave energy from any direction. The output power of EMG and TENG reaches 6.7 mW and 8.01 μW, respectively, at the wave driving frequency of 1.4 Hz. The TENG can effectively harvest the energy from the wave and supply power to wireless sensor nodes of the monitoring system. Thus, this system realized the remote transmission of marine information and real-time monitoring of the marine environment. At last, the self-powered wireless sensing node transmission system was tested in a laboratory and in the sea. It turns out that the self-powered hydrophone system realizes sustainable acoustic signal sensing and has important applications in long-term synchronous ocean monitoring.

### 3.2. Human Monitoring

Recently, multifunctional sensors have been widely used in human movement and physiological monitoring, which can detect dangerous conditions and human health problems in order to provide help in a timely manner. Due to the advantages of the TENG, self-powered wireless sensors based on the TENG play more and more important roles in human movement and physiological detection. 

#### 3.2.1. Human Movement Measurement

In recent years, a large number of flexible sensors have been used to detect human motion [70,71,72,73,74]. Meanwhile, many textile sensors are used to monitor human motion with the development of wearable technology [75,76,77].

The pressure and strain sensor has the advantages of high sensitivity and diversified functions. It can convert various forms of external stimuli such as human movement into electrical signals, given its broad application prospects in monitoring human health and motion [78]. In recent years, in the field of daily health monitoring and robot research, the pressure and strain sensors based on the TENG have received extensive attention due to their simple structure and self-powered characteristics [79,80,81,82].

**Figure 5:**
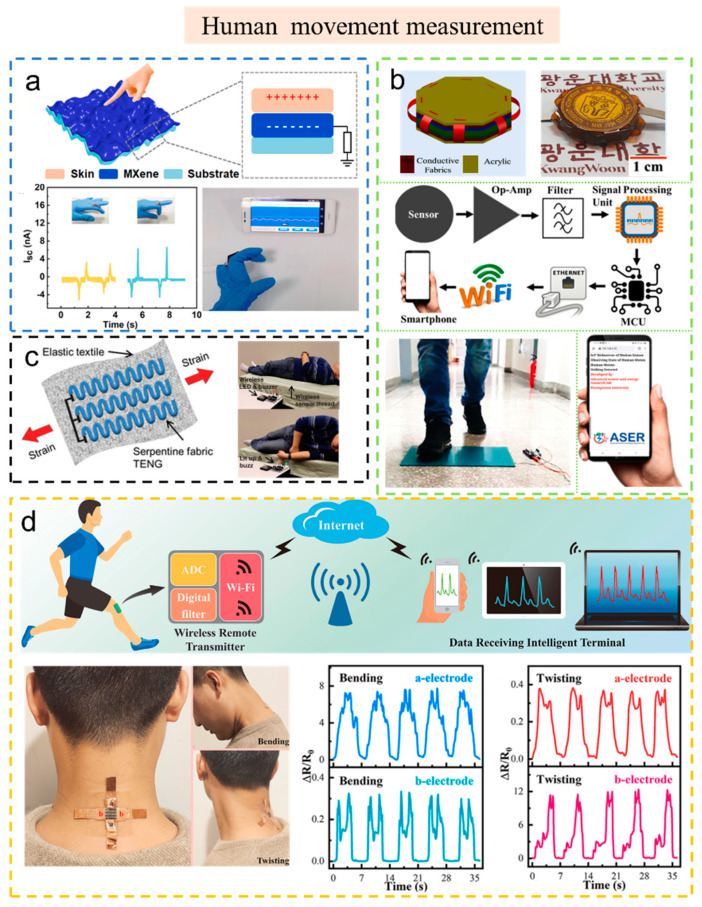
The self-powered wireless sensors and systems based on TENG used in human movement measurement. (**a**) Highly sensitive self-powered pressure and strain sensor based on crumpled MXene film for wireless human motion detection. Reproduced with permission [78]. Copyright 2022, Elsevier Ltd. (**b**) Cation-functionalized nylon composite nanofibrous mat as a highly positive friction layer for robust, high-output triboelectric nanogenerators and self-powered sensors. Reproduced with permission [83]. Copyright 2021, Elsevier Ltd. (**c**) Single-thread-based wearable and highly stretchable TENGs and their applications in cloth-based biomedical sensing. Reproduced with permission [84]. Copyright 2016, John Wiley & Sons. (**d**) Self-powered and multi-mode flexible sensing film with patterned conductive network for wireless monitoring in healthcare. Reproduced with permission [85]. Copyright 2022, Elsevier Ltd.

Cao et al. reported a stretchable TENG [78], which was fabricated by brushing uniform MXene ink on an inflated latex balloon to obtain a high-tensile MXene film. The stretchable TENG has very good tensile properties: a maximum areal strain ratio of 2150% and a linear tensile ratio of 400%. The sensor based on this TENG exhibits excellent sensitivity of 2.35 V kPa^−1^ under pressures ranging from 0.3 to 1.0 kPa. As shown in Figure 5a, the sensor is successfully integrated into the wireless motion monitoring system, which can feed back the human motion state to a smartphone in real time with an independent power supply, indicating that it has broad application prospects in the field of wearable sensors. 

Figure 5b shows a TENG with new positive triboelectric material and a high electrical output performance (*V*_OC_ of 380 V and *I*_SC_ of 80 μA) [83]. The TENG can be used as a self-powered pressure sensor with high performance (sensitivity of 1.01 V kPa^−1^ and linearity over a large pressure range of 0.2 to 36 kPa). This TENG is also used as a self-powered motion sensor for monitoring real-time human motion such as slow walking, brisk walking, jogging, running, and jumping. 

A wireless patient-care system based on a single-thread TENG is shown in Figure 5c [84]. The single-thread TENG can harvest energy via contact between the silicone rubber-coated stainless-steel thread and the skin. The nanogenerator can be stitched into a bedspread to monitor human motion as a smart bed. When the patient moves toward the bedside, the system will send a warning signal by a buzz or LED light to warn them.

In addition to textile sensors, other flexible sensors are also widely studied and applied. Zhu et al. fabricated a TENG consisting of a C-AgNW/TPU conductive film and a polytetrafluoroethylene (PTFE) film [85]. Moreover, they designed a self-powered and multi-mode flexible sensing system, which contained a TENG, flexible solid-state supercapacitors (FSSC), a strain sensor, a wireless transmitter, and intelligent terminals (Figure 5d). The system can realize remote real-time monitoring of human health. They attached the C-AgNW/TPU sensor to the back of the neck and monitored various neck activities. The as-fabricated strain sensor could aptly detect human activity, and the authors proposed the sensor be applied to rehabilitation therapy and intelligent robots.

#### 3.2.2. Human Physiological Measurement

Wearable electronics can monitor the health status of individuals accurately and continuously in real time [86,87,88,89]. Flexible sensing devices can be also used for human physiological monitoring.

**Figure 6:**
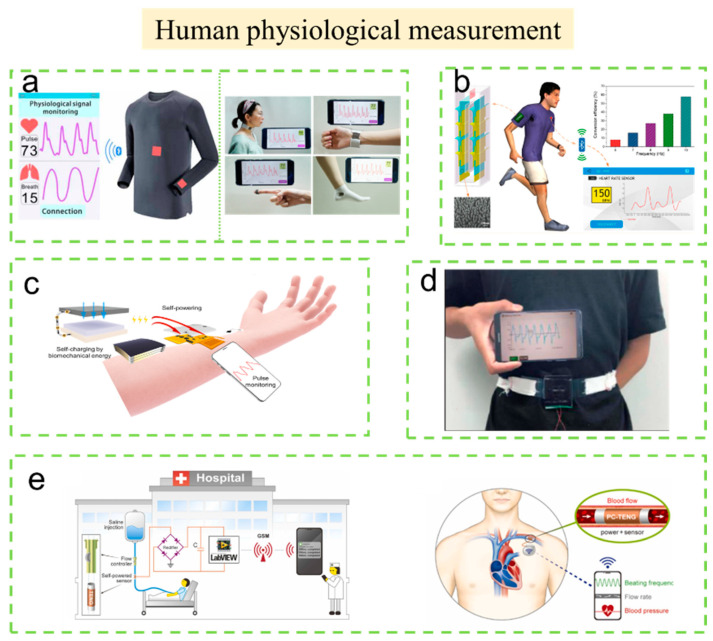
The self-powered wireless sensors and systems based on TENG used in human physiological measurement. (**a**) Machine-knitted washable sensor array textile for precise epidermal physiological signal monitoring. Reproduced with permission [90]. Copyright 2020, American Association for the Advancement of Science. (**b**) A TENG-enabled body sensor network for self-powered human heart-rate monitoring. Reproduced with permission [91]. Copyright 2017, American Chemical Society. (**c**) A self-powered triboelectric MXene-based 3D-printed wearable physiological biosignal sensing system for on-demand, wireless, and real-time health monitoring. Reproduced with permission [92]. Copyright 2022, Elsevier Ltd. (**d**) Waist-wearable wireless respiration sensor based on triboelectric effect. Reproduced with permission [93]. Copyright 2019, Elsevier Ltd. (**e**) Flowing water-based tubular triboelectric nanogenerators for sustainable green-energy harvesting. Reproduced with permission [94]. Copyright 2022, Elsevier Ltd.

Heart-rate monitoring plays a key role in personal health management. A triboelectric machine-knitted washable sensor array that is comfortable, efficient, and user-friendly was reported by Fan et al. (Figure 6a) [90]. This all-textile sensor array shows good performance with high-pressure sensitivity (7.84 mV Pa^−1^), a fast response time (20 ms), good robustness (>100,000 cycles), a wide operating frequency range (up to 20 Hz), and a machine-washable nature (>40 cycles). They designed a wireless mobile health monitoring system based on this all-textile sensor array. The system can be integrated into clothing for the real-time monitoring of pulse and respiration signals.

The heart rates of humans at rest and in motion are different. Lin et al. developed a wireless inspection system that can detect the human body in motion (Figure 6b) [91]. The self-powered wireless body sensor network (BSN) system mainly includes five parts: a downy structure-based triboelectric nanogenerator (D-TENG), a power management circuit, a heart-rate sensor, a signal processing unit, and a Bluetooth module. In this system, the D-TENG can harvest the inertia energy of a human walking to power the system. This self-powered system can aptly detect the heart rate during natural walking and transmit the detection results to a mobile phone through wireless transmission. In addition, Yi et al. also reported a self-powered and wireless sensing system, which can realize physiological signal monitoring in real time (Figure 6c) [92]. In this system, the MXene TENG fabricated by the 3D-printing method shows an output power of 816.6 mW m^−2^. Moreover, the sensitivity of this system is 6.03 kPa^−1^, with a low detection limit of 9 Pa and a fast response time of 80 ms. Most importantly, continuous radial artery pulse (RAP) waveform monitoring can be realized without an external power supply.

Respiratory disease is one of the most common human diseases. Real-time monitoring of respiratory status is helpful to detect apnea and provide early warning, diagnosis, and appropriate treatment. As shown in Figure 6d, Zhang et al. developed a waist-wearable wireless respiration monitoring device based on a TENG, which is portable and comfortable and does not affect users’ sleep, daily life, and social activities [93]. In this work, although the exact sensitivity value is not given in the original literature, the accuracy, feasibility, and sensitivity of the device for respiration monitoring are validated via a series of real-time breathing tests on two volunteers with different waistlines and different breathing rhythms. The results show that the respiration monitoring device has high accuracy and sensitivity in the real-time monitoring of respiratory status, which provides a new choice for real-time monitoring of respiratory-related diseases. 

Solid–liquid friction-based TENGs have attracted much attention in recent years. A polytetrafluoroethylene (PTFE)-copper (PC-TENG) tube with a single-electrode mode was fabricated by Munirathinam et al. in 2022 [94]. As shown in Figure 6e, the PC-TENG is used as a self-powered sensor to monitor intravenous (IV) fluid therapy unattended to detect blood pressure or heartbeat in biomedical settings. Both of these applications realize the function of the system of PC-TENG as a self-powered wireless sensor. Due to the high-volume consumption and simple manufacturing technology of PC-TENG, large-scale power generation applications can be realized. The authors believe that this technology will contribute to more advanced sustainable green energy collection technology and self-powered medical equipment.

#### 3.2.3. Emergencies and Rescue

Injuries in emergency settings could be alleviated if the injured is located in a timely manner. Portable self-powered wireless sensors play an important role when disasters happen [95,96,97,98].

**Figure 7:**
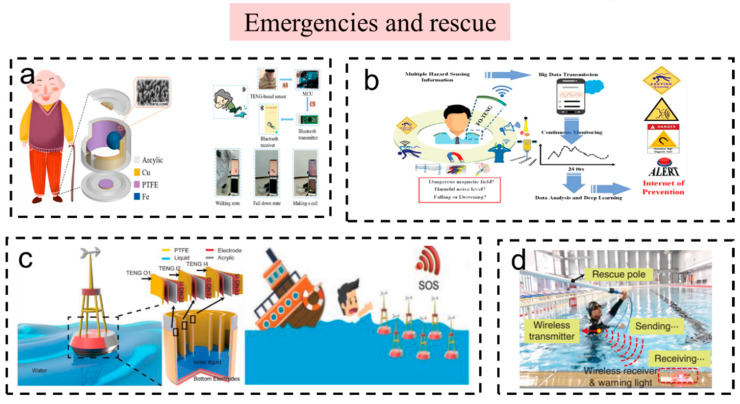
The self-powered wireless sensors and systems based on TENG used in emergencies and rescue. (**a**) Smart Wearable Sensors Based on Triboelectric Nanogenerator for Personal Healthcare Monitoring. Reproduced with permission [99]. Copyright 2021, MDPI (Basel, Switzerland). (**b**) An ultra-shapeable, smart sensing platform based on a multimodal ferrofluid-infused surface. Reproduced with permission [96]. Copyright 2019, John Wiley & Sons. (**c**) A liquid–solid-contact triboelectric nanogenerator and a self-powered wireless save our souls (SOS) system. Reproduced with permission [97]. Copyright 2018, Elsevier Ltd. (**d**) A bionic stretchable nanogenerator for underwater sensing and energy harvesting. Reproduced with permission [98]. Copyright 2019, Springer Nature.

As shown in Figure 7a, Li et al. designed a self-powered smart wearable sensor (SWS) [99] that can accurately and continuously monitor and distinguish various human states, such as stepping, walking, running, and jumping. This system can be used as a remote fall-alarm system for the elderly and patients by sending out a distress signal when they fall down. Ahmed et al. also reported an emergency and rescue system (Figure 7b) [96]. They invented a multimodal ferrofluid-based triboelectric nanogenerator (FO-TENG), which can perceive various dangerous stimuli (a strong magnetic field, noise levels, and falling or drowning) and remind people of the dangers through a wireless transmission system.

When a person accidentally falls into water, it is important to send out a distress signal in time. Li et al. fabricated a liquid–solid-contact triboelectric nanogenerator (LS TENG) and a self-powered wireless save our souls (SOS) system (Figure 7c) [97]. The LS TENGs generate electrical energy stored in a capacitor to drive a wireless SOS radio frequency (RF) transmitter. The system successfully realized a self-powered wireless rescue function. In addition, Zou et al. fabricated a bionic stretchable nanogenerator and an underwater rescue system [98]. The TENG is fabricated by imitating an electric eel in water and constitutes an underwater rescue system with a wireless transceiver module and a warning light. When a person is in danger in the water, the system of the bionic stretchable nanogenerator (BSNG) will work and turn on the distress lamp to seek help (Figure 7d). 

### 3.3. Industrial Production

**Figure 8:**
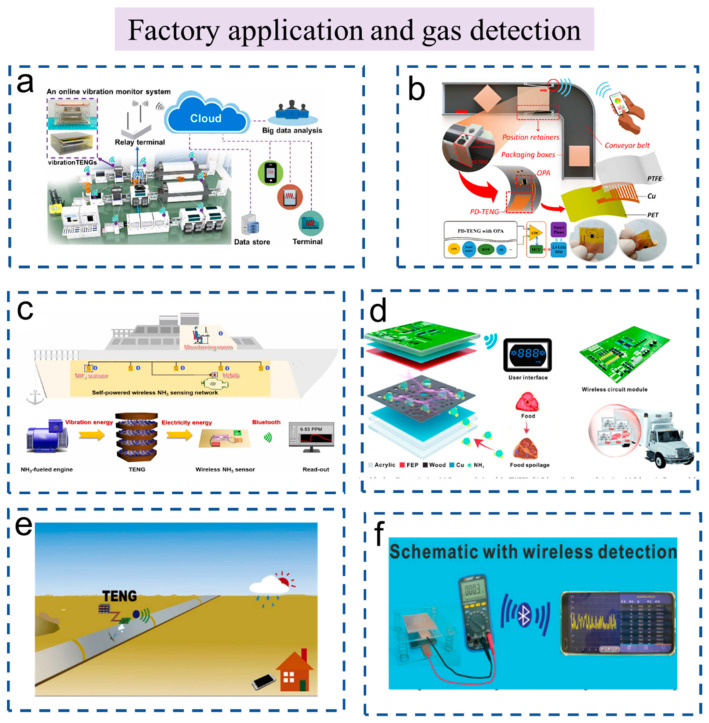
The self-powered wireless sensors and systems based on TENG used in industrial production. (**a**) Broadband vibration-energy-powered autonomous wireless frequency monitoring system based on triboelectric nanogenerators. Reproduced with permission [102]. Copyright 2022, Elsevier Ltd. (**b**) Triboelectric nanogenerator-based wireless sensing for food precise positioning. Reproduced with permission [3]. Copyright 2022, Elsevier Ltd. (**c**) A full-set and self-powered ammonia leakage monitor system based on CNTs-PPy and triboelectric nanogenerator for zero-carbon vessels. Reproduced with permission [107]. Copyright 2022, Elsevier Ltd. (**d**) Integration of a porous wood-based triboelectric nanogenerator and gas sensor for real-time wireless food-quality assessment. Reproduced with permission [108]. Copyright 2021, Elsevier Ltd. (**e**) The CO gas sensing system based on Sub-1G communication. Reproduced with permission [109]. Copyright 2020, Elsevier Ltd. (**f**) Wireless detection system for NH3. Reproduced with permission [110]. Copyright 2022, John Wiley & Sons.

As modern industry develops rapidly, mechanical equipment has been playing an important role in industrial production. If equipment operation is abnormal, it not only causes property damage but also causes safety hazards. Therefore, the normal operation of detection equipment is very important in industrial production [100,101]. Figure 8a shows a smart factory reported by Zhang et.al. They designed an autonomous Wireless Frequency Monitoring System (AWFMS) driven by broadband vibration energy based on two TENGs [102]. In this system, one of the TENGs is used for harvesting energy and the other is used for sensing. The TENG for broadband vibration energy harvesting (P-TENG) has a high open-circuit voltage in the range of 200 V to 380 V when the vibration frequency is between 6 Hz and 20 Hz. The highest output power is above 8.8 mW when the vibration frequency increases from 6 to 20 Hz. Another TENG is used for active vibration frequency sensing (S-TENG) and has excellent stability with over 36,000 operating cycles, which shows the great application potential of this system for frequency monitoring. The AWFMS has implemented self-powered and autonomous vibration monitoring of the equipment in a factory. This research shows the extensive application and broad prospects of TENGs in the Internet of Things, big data, intelligent factories, and other fields.

Yang et al. designed a TENG position sensor for conveyor systems (Figure 8b) [3]. The position sensor unit (PD-TENG) is obtained by directly printing the integrated TENG electrode and copper circuit on the copper layer Polyimide (PI) film using a nanosecond laser, coating the polytetrafluoroethylene film on the electrode, and welding the chip operational amplifier and resistance to the copper circuit. The system can locate objects accurately, which is comparable to the photoelectric sensor. However, the system is far less fake than the photoelectric sensors. What is more, the system can also be used for goods counting. The self-power wireless sensor system shows good application prospects in the industrial field.

Food deterioration can produce some toxic gases. In the factory operating environment, combustible gases (steam) and toxic gases (steam) are often produced due to leakage, volatilization, or other reasons [103,104,105,106]. Figure 8c shows a self-powered full-set ammonia leakage monitoring device and system [107]. This complete system is composed of a TENG, an ammonia-sensing system, and a signal-collecting and -transmitting system. The TENG is installed on the engine of the ship and harvests energy to power the whole sensing system. The ammonia-sensing system can detect ammonia with a low detection limit (0.2 ppm), a short response time (approximately 90 s), good selectivity, good stability, and low cost. It reveals the potential application value of the sensor in monitoring ammonia gas under different conditions.

Real-time monitoring of perishable food is essential to reduce social costs and foodborne diseases. Cai et al. designed a self-powered TENG-based wireless gas sensor system (TWGSS) (Figure 8d) [108]. The system realizes selective, sensitive, and real-time wireless food-quality assessment in the cold supply chain. The high detection rate of this detection system benefits from the special structure of wood and the high sensitivity of the carbon nanotube materials for ammonia. The TENG exhibits an ammonia-sensing response of 0.85 at 500 ppm with the signals transferred to the user interface. 

Liu et al. designed a wind-driven self-powered wireless environmental sensing system (Figure 8e) [109]. The CO gas concentration monitoring system can automatically transmit data within 1.5 km through Sub-1G every 18 min. The system can be used as a remote leak alarm for the gas pipeline. Zhang et al. developed a real-time self-powered gas-sensing system based on a gas-sensitive cellulose nanofibril (CNF) triboelectric material (Figure 8f) [110]. The system can accurately identify the change in ammonia concentration in the range of 10–20 ppm and transmit the signal wirelessly to the user interface to achieve online real-time monitoring of NH_3_ in the environment.

### 3.4. Daily Life 

With the development of society, the emergence of the Internet of Things has promoted the intellectualization of people’s lives. As an important part of the Internet of Things, wireless sensors based on TENGs play an important role in improving human life.

#### 3.4.1. Auxiliary Device

**Figure 9:**
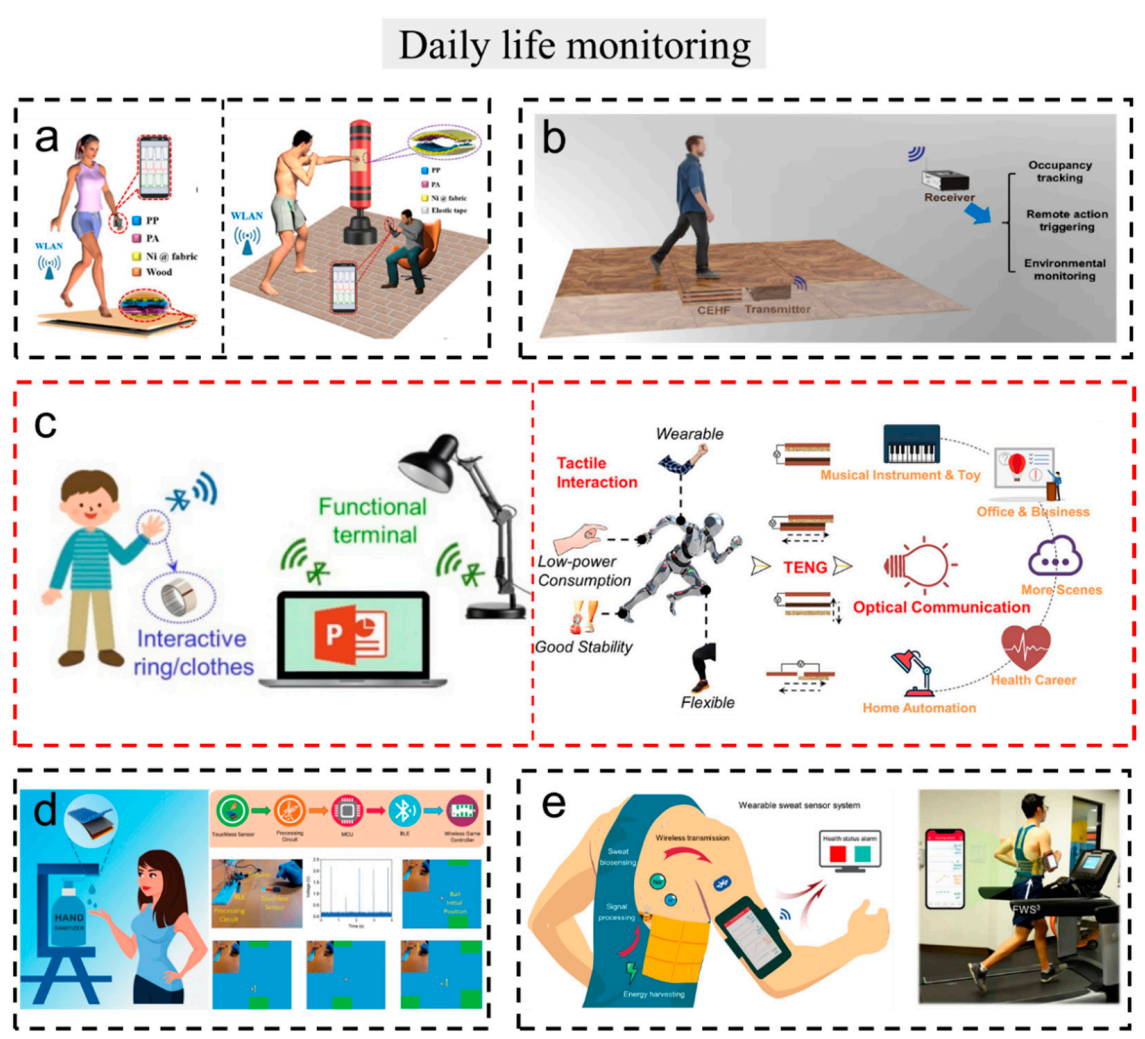
The self-powered wireless sensors and systems based on TENG used in daily life. (**a**) A system is used to collect pedestrian volume and monitor pugilism training. Reproduced with permission [111]. Copyright 2019, Elsevier Ltd. (**b**) Energy-harvesting floor from commercial cellulosic materials for a self-Powered wireless transmission sensor system. Reproduced with permission [112]. Copyright 2021, American Chemical Society. (**c**) A universal and arbitrary tactile interactive system based on self-powered optical communication. Reproduced with permission [113]. Copyright 2020, Elsevier Ltd. (**d**) A self-powered sensor for touchless hand sanitizer. Reproduced with permission [114]. Copyright 2022, John Wiley & Sons. (**e**) Wireless battery-free wearable sweat sensor powered by human motion. Reproduced with permission [115]. Copyright 2020, American Association for the Advancement of Science.

Various sensors are used for intelligent life to help people live more conveniently. Figure 9a displays a fabric-based triboelectric nanogenerator (FB-TENG), which is composed of polypropylene non-woven fabrics (PP-NWF), commercial polyamide 66 fabrics (PA-66-F), and conductive fabrics [111]. The system was used for collecting pedestrian volume. When a person steps on the FB-TENG, a peak will be displayed on multiple clients in real time wirelessly. In addition, a self-powered pugilism training monitor is designed with the system. The work demonstrated potential applications including self-powered motion tracking, tactile sensing, and remote wireless training monitoring systems.

#### 3.4.2. Smart Home Applications

A smart house is a living environment that people are pursuing. The smart home is becoming more and more possible with the development of science and technology. Gu et al. invented a commercial cellulosic material-based energy-harvesting floor (CEHF) with a high output performance and a real-time-powered wireless transmission sensor system (Figure 9b) [112]. The system can be directly installed or integrated with conventional floor products, effectively converting human movements into electrical energy. Even when soaked in water, it shows good durability and stability. In addition, the system can instantaneously power a wireless transmission sensor system with a 1:1 step-to-signal (transmit and receive) ratio.

Intelligent life in the home cannot be separated from various home electronic devices. Huang et al. invented a universal and stable tactile interactive system (TIS) on arbitrary objects through a self-powered optical communicator triggered by triboelectric signals (Figure 9c) [113]. This work is for the first to successfully convert external mechanical energy into electrical energy through a TENG and then convert it into a digital signal through an LED-photoresistor optical communicator and bleeder circuit. This system successfully realized wireless passive control of various household electronic devices. 

#### 3.4.3. Convenient Equipment

With the spread of various popular viruses in society, contactless equipment has become an indispensable device. Kumar et al. proposed a Siloxene/Ecoflex nanocomposite-based high-performance contactless TENG and contactless system device (Figure 9d) [114]. In order to improve the overall output performance of TENG, sandpaper templates are used to form microstructures and molybdenum disulfide (MoS_2_) incorporated with laser-induced graphene (LIG) as a charge trapping interlayer. The device was successfully demonstrated as a self-powered sensor for non-contact hand sanitizer. When dry hands are placed under the device, no hand sanitizer will flow out. However, when wet hands are placed under the device, hand sanitizer will flow out.

#### 3.4.4. Exercise Equipment

In today’s life, the living standard of human beings has been improved, and exercise has become an effective way for human beings to amuse themselves and maintain their vitality. Song et al. proposed a novel TENG (Figure 9e) [115], which has highly robust, mass-producible, and battery-free wearable characteristics. The authors also designed a battery-free triboelectric drive system, which can supply power for multiple sweat biosensors and transmit data wirelessly to the user interface through Bluetooth during human exercises.

## 4. Summary and Perspective

In summary, we have reviewed the applications of self-powered wireless sensors based on TENGs in environmental monitoring, human monitoring, industrial production, and daily life. It can be found that self-powered wireless sensors and systems based on TENG show promising applications in the future [116].

In the environmental monitoring field, self-powered wireless sensors based on TENG can detect wind speed, rainfall intensity, humidity, etc. in the environment in real time, which has replaced human observation and provides great convenience to people. In the human monitoring field, self-powered wireless sensors based on TENG can monitor certain conditions of the human body and physiology in real time, so people can better determine their physical conditions and live a better life. Furthermore, some systems can detect hazards and call for help in various ways. In the industrial production field, self-powered wireless sensors and systems based on TENGs help human beings to build intelligent factories and detect certain toxic gases, thus protecting human life and property. In daily life, self-powered wireless sensors and systems based on TENGs help people to build smart homes and smart places for intelligent life. Presently, self-powered wireless sensors will provide great convenience to human beings.

**Figure 10:**
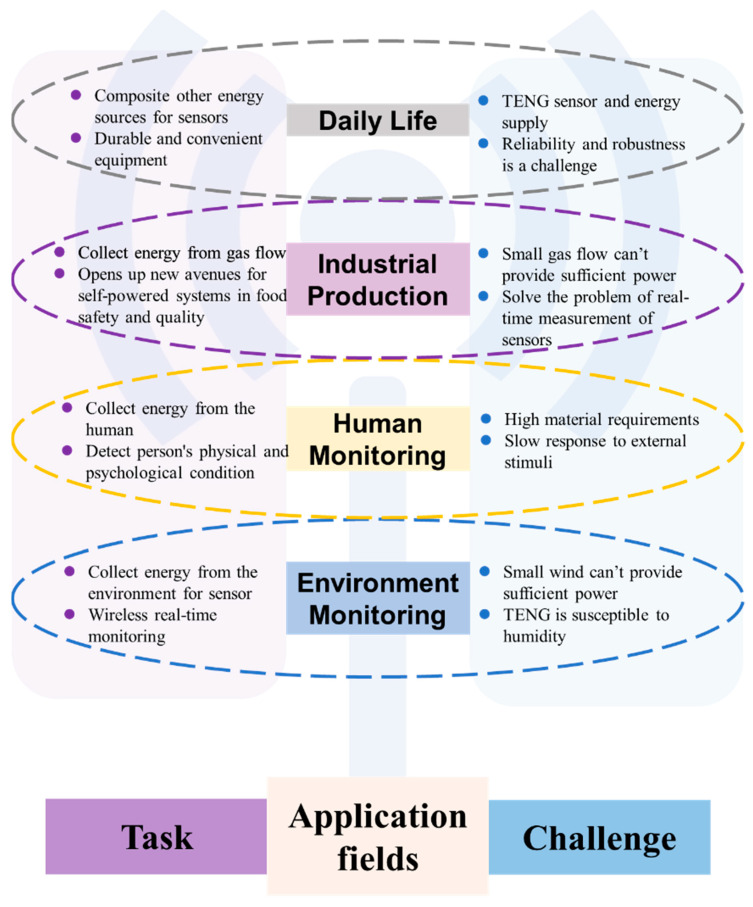
Summary and perspectives of self-powered wireless sensors based on TENG applications in environment monitoring, human monitoring, industrial production, and daily life.

Although self-powered wireless sensors and systems based on TENGs have indeed promoted the development of monitoring in the environment, human health, industrial production, and daily life, there are still some challenges that need to be addressed (Figure 10). 

### 4.1. TENGs as the Power sources

Regarding output performance, it is currently still challenging to obtain high output performance of TENGs. With the development of theoretical research, it has been proved that advanced friction materials and corresponding structural design are a promising direction to obtain high output performance. The other direction is to optimize the coupling between TENG and the external environment for better energy collection efficiency.

Concerning durability, the TENG is widely used to harvest mechanical energy from the environment. Due to the working mechanism of TENGs, long-term repeated friction will reduce the performance and life of TENGs to some extent. Therefore, determining how to prolong the service life of devices should be considered in the process of device preparation and design without affecting the device output.

In terms of power management, the power management circuit is a critical unit to achieve a continuous and stable power supply. Nevertheless, owing to the high voltage and low current characteristics of the TENG, existing conventional commercial power management circuits do not match well with the output of TENG. Hence, it is important to create a more compatible power management circuit for the TENG.

Furthermore, regarding hybrids, for better mechanical energy harvesting, TENGs are commonly used in combination with other types of nanogenerators. The matching efficiency between two (or more) different nanogenerators still needs to be improved in order to achieve effective mixing.

### 4.2. TENGs as the Sensors

In regard to sensitivity, excellent sensitivity has always been a challenge when developing wireless sensors. The output performance requirements listed above also apply to this problem. In addition, new structures can also be used to improve sensitivity.

Concerning stability, stability is not as important for sensors for qualitative analysis, but it is one of the most important performance characteristics of sensors for quantitative analysis. Some special environments have a great impact on a TENG (such as humidity), and certain designs to maintain environmental stability are necessary.

In terms of multifunctionality, multifunctional sensors can reduce the volume and energy consumption of self-powered wireless sensors. Therefore, this is also a direction for the future development of wireless sensors.

## Data Availability

The data presented in this study are available upon request from the corresponding author.
